# Dietary intake of polish organic and conventional fruit growers and their life partners – a pilot study

**DOI:** 10.3389/fpubh.2024.1345402

**Published:** 2024-04-15

**Authors:** Hubert Dobrowolski, Bartosz Szumigaj, Dariusz Włodarek, Renata Kazimierczak, Justyna Obidzińska, Ewa Rembiałkowska

**Affiliations:** ^1^Department of Functional and Organic Food, Institute of Human Nutrition Sciences, Warsaw University of Life Sciences (SGGW), Warsaw, Poland; ^2^Department of Dietetics, Institute of Human Nutrition Sciences, Warsaw University of Life Sciences (SGGW), Warsaw, Poland

**Keywords:** organic food, diet, dietary intake, fruit growers, orchardists, farmers, needs fulfillment

## Abstract

Diet is one of the elements that contribute to health and quality of life. There are significant discrepancies between the diets of people living in different regions, with different beliefs, or with different approaches to sustainability and ecology. There is a lack of research on dietary intake among organic and conventional fruit growers. The aim of our study was to examine the diets of orchardists and their immediate life partners in terms of meeting energy requirements, nutrient intake and fulfillment of dietary recommendations in this group. Fifty-three participants (28 in the organic group and 25 in the conventional group) took part in the study. Dietary data were obtained using the 3-day dietary record. Body mass and height were measured and BMI was calculated. Physical activity was estimated using a questionnaire method. The study group was aged 44 ± 8 years, with a body weight of 84 ± 16 kg and a height of 172 ± 9 cm. The mean BMI was 28 ± 4 kg/m^2^. Mean energy intake with diet was 2170 ± 606 kcal/day with needs of 3002 (1991–5144) kcal/day. A significant proportion of the study group did not fulfill their calcium and vitamin D requirements. In addition, a significant proportion of the conventional fruit growers did not cover their needs for potassium, magnesium and vitamins: E, C, and folate. Both groups had too high an intake of cholesterol and saturated fatty acids, and too low an intake of polyunsaturated fatty acids. In conclusion, the orchardists' diets mostly provided adequate amounts of nutrients, with inadequate intakes of calcium, vitamin D, cholesterol, and fatty acids. The diets of organic fruit growers were significantly richer in selected nutrients.

## 1 Introduction

Diet is a factor that undoubtedly affects human health. Many studies have linked poor diet with the non-communicable disease (NCD) (1). Adequate intake of nutrients and adherence to nutritional standards is an effective source of prevention of these diseases and helps to maintain optimal health (2). Estimating the intake of individual nutrients in the food ration is therefore crucial for estimating the risk of selected diseases in specific population groups, as well as for implementing preventive measures to improve the quality of the diet and ensure the wellbeing of members of these groups.

Diet and nutrition are influenced by a number of factors, such as financial situation, occupation or household management. The specific nature of the work performed by farmers and fruit growers can affect more than just their diet. These individuals will be characterized by their specific quantity and frequency of food consumed as well. Due to the often limited time or opportunities to eat a meal (e.g., working in the middle of an orchard without the possibility of a break) also the quality of the diet and the frequency of consumption of particular food groups may differ significantly from the general population. However, only a few studies evaluating the diet of this group of people are available so far. From the few observed in the literature, it can be concluded that their diet is based on flour, bread, pasta (3, 4), milk, eggs, fruit and vegetables and meat (3), but with less beef consumed compared to other population groups (5). Other studies have shown that people involved in food production are more likely to eat the food they produce (6). It is therefore necessary to examine the diet of fruit growers, as the available data indicate that it may deviate from the regular model. On the other hand, ensuring an adequate intake of nutrients with the diet is a key to maintaining health. Meeting the nutritional needs of fruit growers is therefore important from a public health perspective, due to their special character.

A specific way of cultivation, orcharding and horticulture is organic methods. It is regulated at EU level by Regulation (EU) No 848/2018 of the European Parliament and of the Council on organic production and labeling of organic products and repealing Council Regulation (EC) No 834/2007 (7) and at national level by Act of June 23, 2022 on organic farming and organic production (8). The organic farming system implies a ban on the use of synthetic pesticides, feed enrichment, the minimization of veterinary drugs and takes a restrictive approach to the use of food additives in the products. The outcome is supposed to be both the protection of nature and the environment, as well as obtaining a food product of high quality and that is safe for the consumer. This requires more work from orchardists, which has an impact on the reduction of free time including time spent preparing and eating meals. At the same time, the autumn-winter season reduces the workload associated with cultivation which can also affect nutrition. Significant differences between organic and conventional orcharding can therefore also have a significant impact on diet and nutrient intake with the food ration. Learning about the differences between orchardists who manage the orchard using different methods will make it more possible to adapt the dietary recommendations for this group. It can be assumed that organic fruit growers will be more likely to consume organic food produced by themselves (6) or from other organic farms. Greater consumption of self-produced organic foods may also be due to a strong confidence in their pro-environmental benefits, and as polish research indicates organic farmers present a higher level of ecological awareness than their conventional colleagues, both in central and eastern Poland (9, 10).

According to studies, organic food tend to be richer in selected nutrients, such as antioxidants (11–16), n-3 polyunsaturated fatty acids (17–19), or CLA (19). Consumption of organic food can therefore also result in a higher intake of selected nutrients that not only have been shown to have health-promoting properties, but are often consumed in insufficient quantities. So far, research indicates that organic consumers living in cities consume a better balanced diet that is more in line with nutritional recommendations (20, 21). However, there have been no studies to date on diet and food consumption with conventional and organic farmers and fruit growers. Learning about these values and the differences between groups is an important issue from a public health perspective.

Therefore, the aim of this study was to examine the diets of orchardists and their immediate life partners in terms of meeting energy requirements, nutrient intake and fulfillment of dietary recommendations. This study also included a comparison of differences between organic and conventional fruit growers living in the immediate area.

## 2 Materials and methods

The study was carried out in autumn-winter period, when no intensive work is carried out in the orchards. This period is characterized by negligible activity of orchardists in agricultural work. The number of activities of an exertional nature is limited, and consequently the physical activity rate is lower. Associations of organic fruit growers were used to recruit the study group. Belonging to such an association guaranteed a high level of involvement in the way orchards were run, as well as the homogeneity of the group. In addition, organic fruit growers were asked to identify other orchard owners potentially interested in participating in the study. The group of conventional fruit growers was recruited based on the location of their residence and orchard - the group was selected to reside and operate in the immediate vicinity of the group of organic fruit growers. This procedure ensured similar environmental conditions, similar distance from shops and access to food and groceries, which guaranteed a reliable comparison of diet between the groups. Recruitment to the study group was therefore done using the purposive selection method and then followed up with the snowball collection method.

Participants were included in the study if they had been in the orchard business for at least 5 years or were the partner of such a person. An additional criterion for inclusion in the study was age between 20 and 65 years. Participants who were chronically ill or had been on sick leave for the past 2 months preventing them from working on the orchard farm were excluded from the study.

A total of 53 participants took part in the study. Twenty-eight participants were from an organic orchard (16 orchardists and 12 partners) and 25 participants were from a conventional orchard (15 orchardists and 10 partners) living in the immediate neighborhood. The partners of the fruit growers are characterized by similar lifestyles and physical activity, due to their assistance with the daily chores, as well as having a similar world view on environmental issues and a similar diet, resulting from meals eaten together as a family. The number of participants in the study was considered sufficient given the number of organic farms in Poland, as well as the pilot nature of the study to assess the need for more extensive research on this population, as well as to determine the adequacy of the research methods used.

Approval for the study was obtained from the Rector's Committee on Ethics in Human Research at SGGW (decision no. 7/RKE/2023/U of 30 January 2023). All study participants were informed about the study, the measurements taken, the data collected and the use of the information collected during the study. All questions and concerns of the study participants were answered in detail and comprehensively by the research team carrying out the measurements. Written consent was obtained from each participant for participation in the study and in any research procedures.

All study participants underwent anthropometric measurements. Participants in the study were measured twice for height and weight. If the results of the measurements differed significantly from each other, another (third) measurement was taken and then two measurements close to each other were used for further analysis. An arithmetic mean was drawn from the two measurements. Body height was measured using a SECA 213 stadiometer. Measurements were taken without shoes or socks. The head arrangement corresponded to the Frankfurt plane position. Body weight was measured using a Tanita BF-350 device. Estimated clothing weights were subtracted from the results obtained.

Dietary intake data were performed using the 3-day food recording method. The 3-day food record questionnaire is commonly used in research to determine the quality and composition of diet and the fulfillment of nutritional needs. This method allows a reliable and accurate estimation of the intake of almost all nutrients (22). The recording of the products consumed took place on 3 days - 2 working days and 1 day free of work tasks. Participants were carefully instructed on how to complete the questionnaire, the need to accurately report the composition and recipe of the food and all products consumed and how to accurately record the amount of food consumed. The way in which participants were instructed guarantees high data quality and high reliability of the results obtained. National standards (23) were used as a reference for the recommended values. EAR norms were used to estimate the realization of nutritional needs and, if this norm was not established for a nutrient, the sufficient intake level of AI was used. Questionnaires were distributed at meetings where anthropometric measurements were taken. Participants were asked to complete the questionnaires in the coming week to enable dietary analysis based on the measurements taken. After completing the note-taking questionnaires, participants provided the diets to the research team. If necessary, participants were asked to clarify the information in the questionnaire to prevent possible errors.

A questionnaire created by Johansson and Westerterp (24) was used to determine the Physical Activity Level (PAL). The questionnaire provides a simple way to estimate physical activity rates according to the intensity of activity during work and leisure time. The questionnaire has previously undergone a validation procedure using a double-labeled water method, which proves its accuracy. The Harris and Benedict formula was used to determine Resting Energy Expenditure (REE) (25). The Total Energy Expenditure (TEE) was estimated by multiplying the REE and PAL results.

Statistical analysis was performed using SPSS v. 28.0 software (IBM corp.), R and RStudio (The R Foundation). The normality of the distribution was estimated using the Shapiro–Wilk test. For a complete presentation of the data and to make it easier to compare the groups with each other, means with standard deviations, medians as well as minimum and maximum values were presented in tables. Before conducting the main analysis, set of preliminary *t*-tests were done to ensure that there were no statistically significant differences between ages of fruit growers from all of the further compared groups, in order to determine that the variable carries minimal risk of possible confounding of the effects between groups under study. Comparisons between the groups (organic vs. conventional orchardists; man vs. woman; orchardists vs. their partners) were made using the Student's *t*-test in the case of a normal distribution of the data and the *U* Mann–Whitney test in the case of a distribution different from normal. The study's defined significance level was set to α = 0.05.

## 3 Results

The study was conducted on a group of 53 fruit growers with an average age of 44 ± 8 years. The mean body mass was 84 ± 16 kg, while the mean body height was 172 ± 9 cm. The mean BMI in the study group was 28 ± 4 kg/m^2^. No significant differences were observed between the group of organic fruit growers and the group of fruit growers using conventional methods (*p* > 0.05, student's *t*-test). There were also no age differences observed between fruit growers and their partners (*p* > 0.05, student's *t*-test). However, differences were observed between the partners and orchardists groups in height (*p* < 0.001, student's *t*-test) and body mass (*p* < 0.001, student's *t*-test) as well as BMI (*p* = 0.017, student's *t*-test), as a direct result of the gender ratio, with 27 men and four women in the fruit growers' group, and two men and 20 women in the partners' group. However, no differences were observed between organic and conventional fruit growers when comparing fruit growers and partners separately in terms of age, weight and height, and BMI (*p* > 0.05, student's *t*-test). The anthropometric data of the study participants are presented in Table 1.

**Table 1:** Characteristics of the study group.

		**Age**	**Body mass**	**Body height**	**BMI**
		**(mean ±sd**	**(mean ±sd**	**(mean ±sd**	**(mean ±sd**
		**median**	**median**	**median**	**median**
		**min–max)**	**min–max)**	**min–max)**	**min–max)**
Organic	Overall	46 ± 8	84 ± 17	174 ± 8	28 ± 5
		45	87	172	28
		33–63	51–113	161–189	16–36
	Orchardist	47 ± 7	90 ± 13	177 ± 8	29 ± 4
		47	91	179	28
		34–63	65–113	161–189	22–36
	Partner	45 ± 9	75 ± 18	170 ± 6	26 ± 6
		44	70	169	25
		33–62	51–102	161–183	16–36
Conventional	Overall	43 ± 9	85 ± 16	171 ± 10	29 ± 4
		42	83	169	29
		29–63	60–113	154–192	22–38
	Orchardist	44 ± 9	91 ± 16	174 ± 12	30 ± 4
		44	95	173	30
		30–63	64–113	154–192	22–38
	Partner	40 ± 8	75 ± 9	166 ± 5	27 ± 3
		39	75	166	27
		29–57	60–88	161–174	23–34
Overall	Overall	44 ± 8	84 ± 16	172 ± 9	28 ± 4
		44	86	170	29
		29–63	51–113	154–192	16–38
	Orchardist	45 ± 8	91 ± 14	176 ± 10	29 ± 4
		45	95	176	29
		30–63	64–113	154–192	22–38
	Partner	43 ± 9	75 ± 15	168 ± 6	26 ± 5
		43	75	167	26
		29–62	51–102	161–183	16–36

The median REE in the study group was 1682 (1254–2276) kcal/day. At the PAL median 1.7 (1.4–2.3) observed in the study group TEE was 3002 (1991–5144) kcal/day. No statistically significant differences were observed between the group of organic and conventional fruit growers (*p* > 0.05, U Mann–Whitney's test). Orchardists showed statistically significantly higher REE and TEE compared to their partners (*p* < 0.001, U Mann–Whitney's test) and significantly higher physical activity (*p* = 0.037, U Mann–Whitney's test). Energy expenditure and physical activity levels in the study group are presented in Table 2.

**Table 2:** Resting and total energy expenditure and physical activity level in study group.

		**REE**	**PAL**	**TEE**
		**(mean ±sd**	**(mean ±sd**	**(mean ±sd**
		**median**	**median**	**median**
		**min–max)**	**min–max)**	**min–max)**
Organic	Overall	1705 ± 279	1.8 ± 0.2	3065 ± 738
		1690	1.7	3075
		1254–2196	1.4–2.3	1991–4694
	Orchardist	1861 ± 213	1.9 ± 0.3	3458 ± 627
		1896	1.8	3507
		1478–2196	1.5–2.3	2431–4694
	Partner	1496 ± 215	1.7 ± 0.2	2541 ± 525
		1442	1.7	2360
		1254–1932	1.4–2.1	1991–3655
Conventional	Overall	1727 ± 322	1.8 ± 0.2	3052 ± 739
		1587	1.7	2807
		1308–2276	1.5–2.3	2160–5144
	Orchardist	1869 ± 332	1.8 ± 0.2	3361 ± 786
		1995	1.7	3531
		1375–2276	1.6–2.3	2253–5144
	Partner	1514 ± 146	1.7 ± 0.1	2589 ± 310
		1471	1.7	2582
		1308–1810	1.5–1.9	2160–3078
Overall	Overall	1715 ± 297	1.8 ± 0.2	3059 ± 731
		1682	1.7	3002
		1254–2276	1.4–2.3	1991–5144
	Orchardist	1865 ± 272	1.8 ± 0.2	3411 ± 698
		1900	1.8	3531
		1375–2276	1.5–2.3	2253–5144
	Partner	1505 ± 183	1.7 ± 0.2	2563 ± 431
		1465	1.7	2487
		1254–1932	1.4–2.1	1991–3655

REE, Resting Energy Expenditure; PAL, Physical Activity Level; TEE, Total Energy Expenditure.

Table 3 shows the structure of energy and macronutrient intake in the study group. The average energy intake was 2170 ± 606 kcal/day, consisting of an average protein intake of 100 ± 33 g/day, a fat intake of 81 ± 27 g/day and a carbohydrate intake of 270 ± 98 g/day. No statistically significant differences were observed between the groups of organic and conventional fruit growers (*p* > 0.05, student's *t*-test). The majority of study participants did not meet their energy and carbohydrate needs (83% and 89%, respectively). Almost all (94%) participants of the study met their requirements for protein intake (average intake 100.2 ± 32.6 g/day with an average requirement of 61.4 ± 11.9 g/day) and the majority of participants (64%) met their requirements for fat intake (average intake 80.7 ± 26.8 g/day with an average lowest requirement of 68 ± 16.12 g/day).

**Table 3:** Energy and macronutrient intake in the study group.

	**Min–Max**	**Mean ±SD**	**Requirements**	**Percentage below recommendations**
Energy kcal)	1201–4152	2170 ± 606	3059 ± 731	83
Protein (g)	40.6–195.3	100.2 ± 32.6	61.4 ± 11.9	6
Fat (g)	35.6–156.4	80.7 ± 26.8	68 ± 16.12 – 119 ± 28.4^*^	36
Carbohydrates (g)	65.4–543.2	270.1 ± 97.5	435.7 ± 109.6	89

^*^Fat requirements of 20–35% of energy needs.

Tables 4, 5 present the structure of mineral and vitamin intake. A significant percentage of the study group had an insufficient intake of potassium (41.5%) and calcium (69.8%). In the group of organic fruit growers, a significant percentage of participants did not cover their calcium requirements (54%), while in the group of conventional fruit growers, potassium (60%), calcium (88%) and magnesium (52%) were not covered. Participants in the study did not exceed the Upper Level intake (UL). It was observed that the organic fruit grower group consumed significantly more potassium (*p* < 0.001, test t), calcium (*p* = 0.008, U Mann–Whitney's test), phosphorus (*p* = 0.002, student's *t*-test), magnesium (*p* = 0.020, U Mann–Whitney's test), iron (*p* = 0.007, U Mann–Whitney's test), zinc (*p* = 0.015, U Mann–Whitney's test), copper (*p* = 0.011, U Mann–Whitney's test) and manganese (*p* = 0.012, U Mann–Whitney's test) compared to the group of conventional fruit growers. In the case of phosphorus, however, where both groups met the requirement in full, this indicated that the group of organic fruit growers was statistically significantly more likely to exceed the recommendations (*p* = 0.002, student's *t*-test) compared to the group of conventional fruit growers. All participants of the study covered the needs in terms of sodium and phosphorus intake. In addition, all organic orchardists covered the needs in terms of iron, copper and iodine intake.

**Table 4:** Intake and meeting requirements for minerals in the study group.

	**Overall intake**		Needs^*^	**Intake**		*p*–value^**^	**% below requirements**	
	**Min–Max**	**Mean** ±**SD/Median**		**Organic**	**Conventional**		**Organic**	**Conventional**
				**Mean** ±**SD/Median**	**Mean** ±**SD/Median**		**%**	**%**
Sodium (mg)	1923–7326	4531 ± 1327	1500	4517 ± 1189	4549 ± 1491	0.931	0	0
Potassium (mg)	31424–6502	3830 ± 1161	3500	4315 ± 1084	3286 ± 1008	**<** **0.001**	25	60
Calcium (mg)	115, 8–2031	597.2	800–1000	801, 1	546, 0	**0.008**	54	88
Phosphorus (mg)	648.5–2775	1525 ± 506, 4	580	1724 ± 509.8	1304 ± 407.2	**0.002**	0	0
Magnesium (mg)	110.4–747.3	337.3	255–350	364.7	281.0	**0.020**	21	52
Iron (mg)	5.21–46.77	13.82	6–8	14.91	11.68	**0.007**	0	8
Zinc (mg)	6.56–24.38	11.17	6.8–9.4	13.05	9.32	**0.015**	4	16
Copper (mg)	0.46–3.09	1.34	0.7	1.43	1.12	**0.011**	0	4
Manganese (mg)	0.71–12.34	4.42	1.8–2.3	5.06	3.79	**0.012**	4	4
Iodine (μg)	55.91–317.5	174.3 ± 55.08	95	183.1 ± 56.07	164.4 ± 53.32	0.220	0	12

Needs estimated on the basis of Standards for the Polish population (23); Bolded values statistically significant; ^*^Needs (according to EAR / AI) depending on age and gender; ^**^Student *t*-test for normal distribution and the U Mann–Witney's test for a distribution different from normal.

**Table 5:** Intake and meeting requirements for vitamins in the study group.

	**Overall intake**		Needs ^*^	**Intake**		*p-*value^**^	**% below requirements**	
	**Min–Max**	**Mean** ±**SD/Median**		**Organic**	**Conventional**		**Organic**	**Conventional**
				**Mean** ±**SD/Median**	**Mean** ±**SD/Median**		**%**	**%**
Vitamin A (μg)	505.5–22043	1482.0	500–630	1571.6	1473.2	0.285	0	4
Vitamin E (mg)	2.06–38.20	8.98	8–10	9.73	6.18	**0.015**	43	68
Vitamin D (μg)	0.84–24.21	3.93	15	4.53	3.84	0.392	93	96
Vitamin C (mg)	6.16–506.5	87.58	60–75	115.2	70.11	**<** **0.001**	14	52
Vitamin B1 (mg)	0.62–3.21	1.46	0.9–1.1	1.51	1.24	0.113	7	24
Vitamin B2 (mg)	0.80–7.16	1.94	0.9–1.1	2.17	1.69	**0.027**	0	4
Vitamin PP (mg)	8.68–63.43	25.16	11–12	26.56	20.95	0.159	4	4
Vitamin B6 (mg)	0.85–4.42	2.28 ± 0.81	1.1–1.4	2.60 ± 0.71	1.93 ± 0.78	**0.002**	0	16
Vitamin B12 (μg)	1.26–103.3	4.22	2	5.32	3.16	0.050	7	12
Folate (μg)	70.34–695.5	286.0	320	352.0	238.1	**<** **0.001**	39	76

Needs estimated on the basis of Standards for the Polish population (23); Bolded values statistically significant; ^*^Needs (according to EAR / AI) depending on age and gender; ^**^Student *t*-test for normal distribution and the U Mann–Witney's test for a distribution different from normal.

Also, a significant proportion of the study group had insufficient intakes of vitamin E (54%), folate (57%) and vitamin D (94%). Inadequate intake of vitamin E (68%), vitamin D (96%) and vitamin C (52%) was observed in a significant percentage of conventional fruit growers, while inadequate intake in a significant proportion of the group was only observed for vitamin D (93%) in organic fruit growers. Eight people in the study group exceeded the intake of vitamin A of 3000 μg/day, which is the upper intake level (UL) of this vitamin. Of these 8, 6 belonged to the organic fruit grower group and 2 to the conventional fruit grower group. Apart from these cases, the UL level of other vitamins in the study group was not exceeded. In addition, it was observed that the organic orchardist group consumed significantly more vitamin E (*p* = 0.015, U Mann–Whitney's test), vitamin C (*p* < 0.001, U Mann–Whitney's test), vitamin B2 (*p* = 0.027, U Mann–Whitney's test), vitamin B6 (*p* = 0.002, student's *t*-test) and folates (*p* < 0.001, U Mann–Whitney's test), compared to conventional orchardist. Moreover, differences in vitamin B12 intake were on the threshold of statistical significance (*p* = 0, 050001, U Mann–Whitney's test). This was especially important for folates, where organic fruit growers were significantly closer to meeting demand than conventional fruit growers (*p* = 0.003, U Mann–Whitney test). All fruit growers in the organic group covered their vitamin A, B2 and B6 requirements. This was not observed in the conventional fruit growers group.

**Figure 1:**
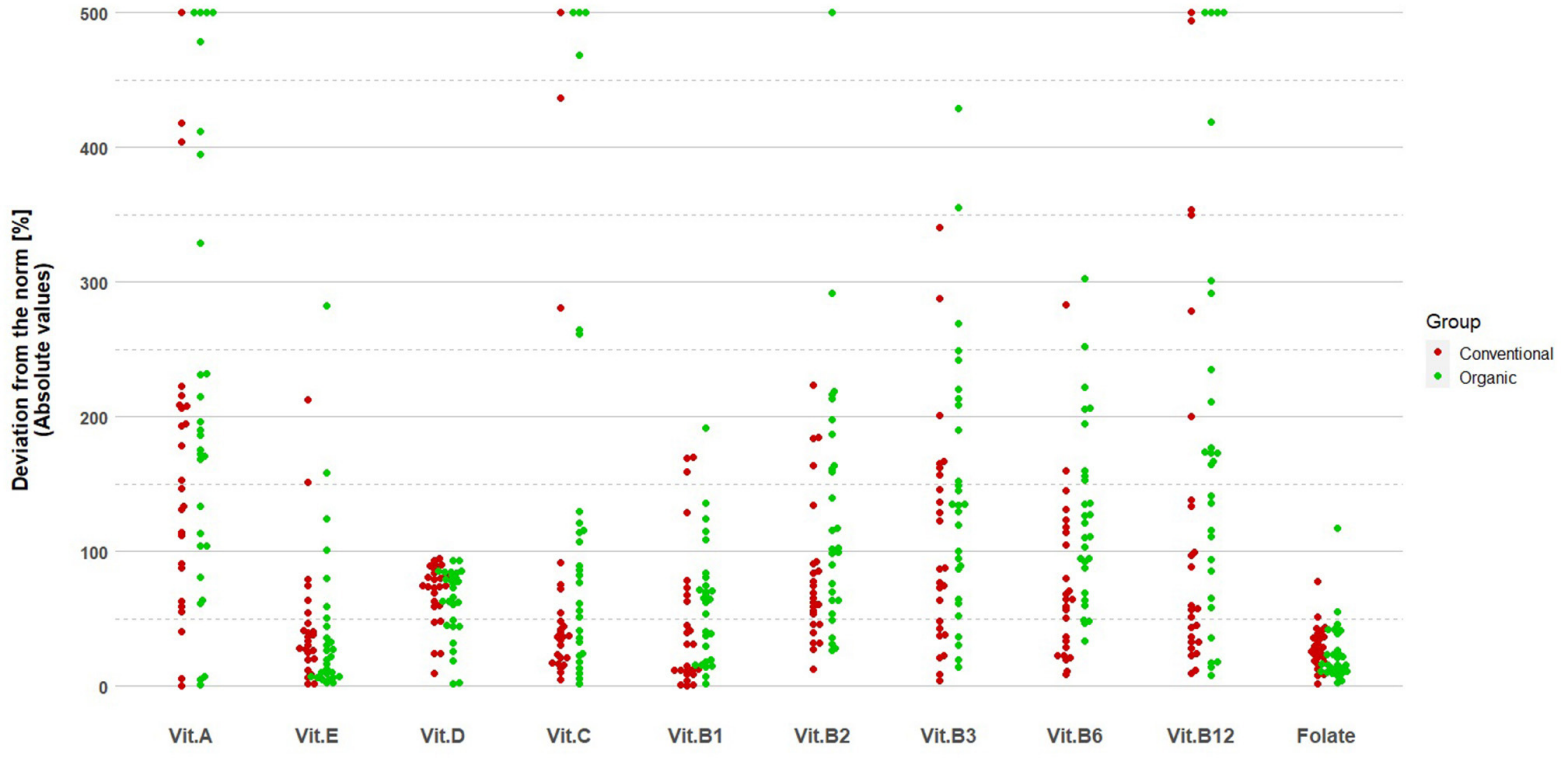
Deviations from the norm in mineral intake among organic and conventional fruit growers.

**Figure 2:**
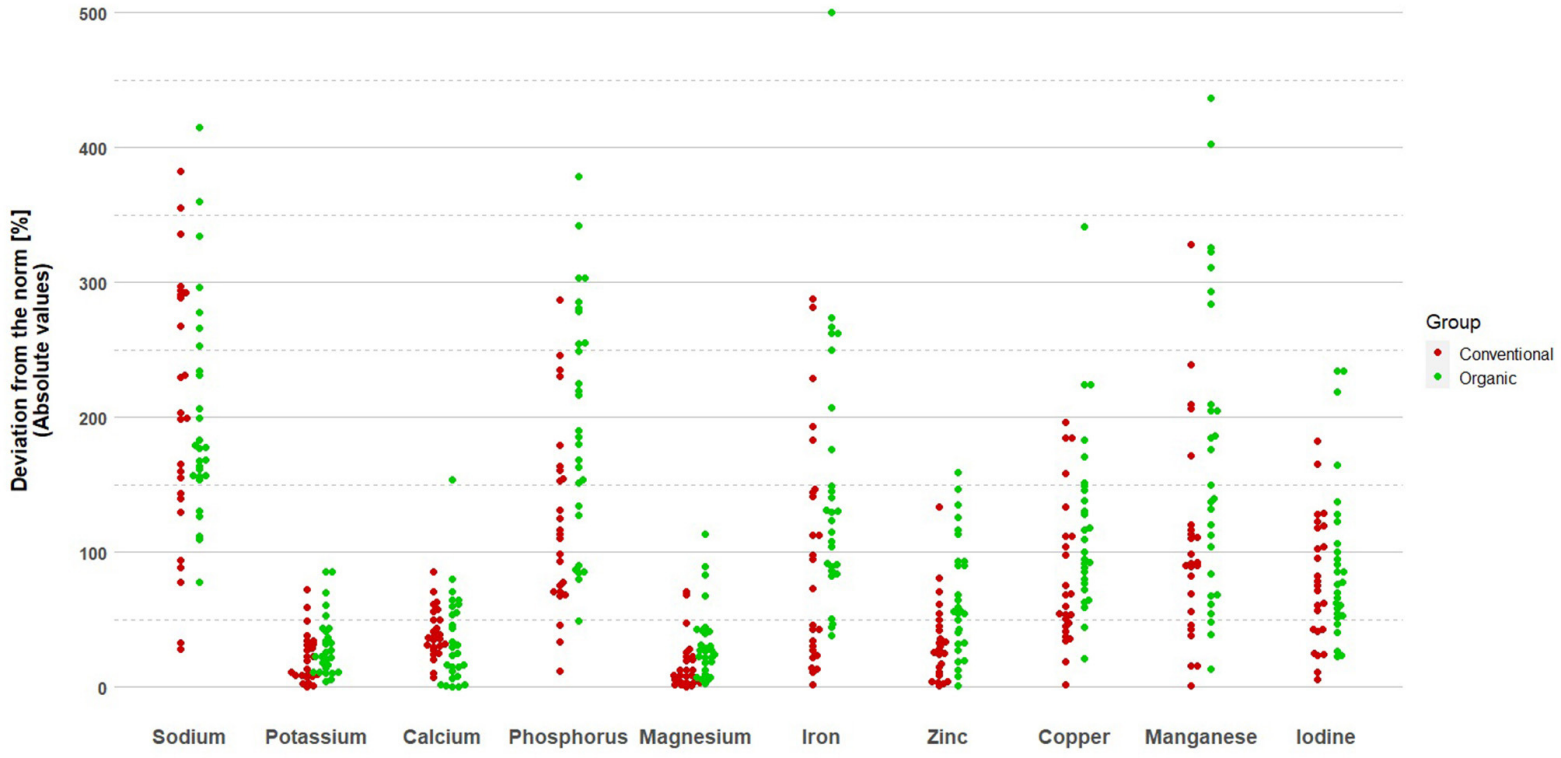
Deviations from the norm in vitamin intake among organic and conventional fruit growers.

Deviations from the fulfillment of mineral and vitamin standards are presented in Figures 1, 2, respectively. The group of organic fruit growers deviated significantly more from the recommended values of intake for phosphorus (*p* = 0.002, student's *t*-test), magnesium (*p* = 0.004, U Mann–Whitney test), iron (*p* = 0.027, U Mann–Whitney test), zinc (*p* = 0.003, U Mann–Whitney test), manganese (*p* = 0.024, U Mann–Whitney test), as well as vitamin B2 (*p* = 0.032, U Mann–Whitney test). The group of conventional fruit growers, on the other hand, deviated more significantly from the recommended values of intake for copper (*p* < 0.001, U Mann–Whitney test) and folates (*p* = 0.003, U Mann–Whitney test).

The mean cholesterol intake in the study group was 465.5 ± 185.4 mg/day. The median intake of fiber in the study group was 20.83 (4.45–48.61) g/day. The median intake of saturated fatty acids (SFA) was 30.64 (13.44–78.85) g/day, while linoleic, α- linolenic and EPA+DHA fatty acids were: 7.77 (4.16–21.22) g/day; 1.28 (0.45–9.55) g/day and 0.13 (0.02–3.41) g/day, respectively. There were no statistically significant differences between groups in the intake of cholesterol, saturated fatty acids, and the fatty acids linoleic, α- linolenic and EPA + DHA (*p* > 0.05, student's *t*-test, U Mann–Whitney test). In turn, orchardists in the organic group consumed significantly more dietary fiber, compared to those in the conventional group (median intake: 26.08 (15.13–48.61) vs. 16.44 (4.45–28.71); *p* < 0.001, U Mann–Whitney test). In the case of dietary fiber, half of the organic orchardists met the minimum requirement for this nutrient, while 92% of conventional orchardists did not. Ninety-two percent of the study group exceeded the recommended maximum cholesterol intake, while 75% of the study group, regardless of orchard method, exceeded the recommended maximum intake of saturated fatty acids. The vast majority of the study group, regardless of production method, also did not consume sufficient amounts of linoleic, α- linolenic and EPA+DHA fatty acids (89%, 62%, and 68%, respectively).

## 4 Discussion

To the best of our knowledge, this is the first study of its kind assessing the quantitative intake of individual nutrients with diets in a group of fruit growers. It is also the first study of its kind comparing the diets of organic and conventional orchardists, and one of the few to address dietary differences between consumers of organic food and consumers of conventionally produced food. Studies on a large French population (30, 000 participants) during the NutriNet-Sante project showed that organic consumers have a better balanced diet, as well as better understanding of what a balanced diet is and its impact on the planet's environment (26, 27). The study therefore makes an extremely important contribution to knowledge about diet and diet composition among those who prefer organic products.

First of all, it should be noted that there were no differences in energy needs and physical activity between the groups of organic and conventional fruit growers. This was due to similar body sizes (no differences in weight and height), age and a similar work mode. The differences in energy expenditure between the fruit growers and their partners were due to the gender disparity between the groups. The group of fruit growers was dominated by men (27 men vs. 4 women) and the group of partners was dominated by women (2 men vs. 20 women). Both Resting Energy Expenditure (REE) and Total Energy Expenditure (TEE) were determined on the basis of body weight and height, which follows directly from the formula (25), and as observed, the partner group showed significantly lower body weight and height. It is therefore natural that they will have a lower energy expenditure. Lower body mass and height are a direct result of the well-known fact that women have a smaller body size and surface area compared to men (28, 29). The lower energy expenditure of women compared to men is also often observed in studies (29–31), so our research is reflected in the current state of knowledge. However, there were no differences in Physical Activity Level (PAL) between orchardists and partners. This may be due to the specific period in which the study was conducted. The orchardists were already spending more time at home, while the partners matched the orchardists in terms of physical activity by participating and helping with the final work of the orchard duties.

The period of the study may also have influenced the lack of differences in PAL between organic and conventional fruit growers. While it is true that the study showed a higher physical activity level between the study group and the general population, which has been observed in other studies (32), the lack of differences between the organic and conventional groups was not expected. Loake (33) observed in her study that the organic farmer experiences a greater level of physical and physiological stress, causing the organic farmer expended more energy and worked at a higher effort intensity than the conventional farmer. In their paper from 2015 Smith et al. (34) indicated that organic farming is more work demanding than conventional farming. The mentioned period of the study resulted in a reduction in work effort in both conventional and organic fruit growers, which may have led to a balancing of work-related activity, which is the main component of physical activity for fruit growers and farmers, and it could result in the observation of no differences in physical activity levels between the study participants.

The average energy intake was 2170 ± 606 kcal/day (with an average requirement of 3059 ± 731 kcal/day), which is too low. As many as 83% of the study group did not have their energy needs met. Prolonged inadequate energy intake can lead to negative health consequences. It should be noted, however, that the best indicator of the fulfillment of energy needs is body mass. A long-term low energy supply with the food ration would imply significantly lower BMI values than those observed in this study, in which the BMI values directly indicated a significant proportion of overweight participants. It is therefore unlikely that the energy value of the ration was so low in the study group. It is likely that this could be due to a transient period in which, exceptionally, the study participants consumed smaller portions, or, more likely, to an underestimation of the size of the portions consumed. Underestimation of energy intake with diet is often observed in studies (35–39). The difference between the energy value of the food ration and the estimated energy expenditure may also be due to an overestimation of the physical activity factor. The period of the survey covered a time when heavy work was severely limited and, consequently, the physical activity of the respondents was limited as well. This, in turn, would have translated into a lower energy expenditure than previously estimated.

Protein and fat were mostly consumed at an adequate level, covering the requirement in a significant percentage of the study group. Only carbohydrates were consumed below the requirement, which resulted in an insufficient energy value of the diet. Carbohydrates are a very important macronutrient for people with a higher daily physical activity than the average person, so an average intake of 270.1 ± 97.5 g/day covering only about 60% of needs is far too low. Carbohydrates are the body's main source of energy, so low carbohydrate intake can severely limit exercise capacity, which can be an important concern in physical occupations. Special attention should therefore be paid to the adequate intake of this macronutrient.

Mineral intake was mostly at a satisfying level in both groups. Only calcium in the organic fruit growers' group and potassium, calcium and magnesium in the conventional fruit growers' group were consumed at insufficient levels. Calcium is responsible for, among other things muscle contraction, enzyme activation, cell differentiation, immune response, programmed cell death and neuronal activity (40), however, its most characteristic feature is its bone and tooth-building function, where it plays a major role (41). Inadequate intake and/or impaired absorption can therefore lead to bone tissue disorders, resulting in the development of osteoporosis (41). This is particularly important in light of the significant phosphorus intake in the study group, where both organic and conventional orchardists significantly exceeded the recommended values. An unfavorable calcium/phosphorus ratio, with additionally insufficient calcium intake, may result in increased resorption of calcium from bones and increase the risk of fractures (42, 43). However, low calcium intake is a problem that has been observed in numerous studies (44, 45).

Insufficient potassium intake was also frequently observed in the group of conventional fruit growers. Potassium is a very important dietary component which, among other things, regulates intra- and extracellular fluid volume, and is also involved in muscle contraction and regulates nerve impulses (46). Adequate potassium intake and an adequate sodium/potassium ratio has been linked to a reduction in the risk of cardiovascular diseases such as stroke and hypertension (47). However, potassium deficiencies in the body are observed very rarely and are mainly associated with diuretics, vomiting or diarrhea (46). It is, nevertheless, advisable to balance potassium intake, as well as the sodium/ potassium ratio, as a preventive measure.

An insufficient intake of magnesium was also observed in the group of conventional fruit growers. Magnesium plays very important roles in the body: regulates various biochemical reactions in the body, including protein synthesis, muscle and nerve transmission, neuromuscular conduction, signal transduction, blood glucose control, and blood pressure regulation, transporting calcium and potassium ions (48). Magnesium deficiency has been linked to a number of diseases, including migraine headaches, Alzheimer's disease, stroke, hypertension, cardiovascular disease and type 2 diabetes (49). However, a low intake of magnesium should not be considered a diagnosis of deficiency, as studies show that many people with too low of an intake had adequate serum concentrations of this element (50). As indicated by population studies, the prevalence of deficiency in populations ranged from 1.7% to 36% (51), therefore, a control examination of serum magnesium concentrations in the study group is advisable.

Vitamin intake in the study group was also mostly at an adequate level. Among the organic orchardists, the inadequate intake was mainly for vitamin D, while among the conventional orchardists it was for vitamins E, D, C, and folate. An inadequate intake of vitamin D, observed in both groups, can result in deficiencies that are associated with common chronic diseases such as bone metabolic disorders, cancer, neuropsychiatric disorders, autoimmune diseases, cardiovascular diseases, and diabetes. However, it is worth noting that the nature of the work of fruit growers is closely linked to being outdoors. The main source of vitamin D in the body is not the diet, but its skin synthesis through UVB radiation. Therefore, it cannot be assumed that too low intake will lead to deficiency and its clinical manifestation. As indicated by a study involving athletes spending time outdoors, the highest serum vitamin D concentrations were observed in late summer and early autumn (52). Given the nature of the work and the start of the study in the autumn season, it can be assumed that serum vitamin D concentrations may have been at an appropriate level. However, given the low calcium intake, routine monitoring for the prevention of osteoporosis seems reasonable. It also seems reasonable to recommend vitamin D supplementation, especially during the study period (autumn-winter), where exposure to sunlight is highly limited (53). Vitamin D supplementation in the Polish population is recommended to prevent vitamin D deficiency (54).

Vitamin E was consumed at insufficient levels by most conventional orchardists. This vitamin is a very important dietary antioxidant (55). However, it is worth emphasizing that, as shown in the NHANES 1999/2000 studies, almost 90% of men and more than 96% of women had vitamin E intakes below the recommended values, while serum deficiency of this vitamin was observed in only 0.5% of the population (56, 57). Deficiencies of this vitamin are extremely rare in the general population and are mainly observed in premature, low birth weight infants and those with digestive and absorption disorders (23). The probability of observing deficiencies in this group is therefore extremely low.

Vitamin C was also consumed at insufficient levels among conventional fruit growers. Adequate intake and maintenance of optimal serum vitamin C concentrations are associated with the prevention of scurvy, coronary heart disease, stroke or cancer. Vitamin C deficiencies have also been linked to increased mortality (58). There is a need to monitor the nutritional status of this vitamin, due to the significant health risks associated with vitamin C deficiency. Recent studies indicate that the problem of vitamin C deficiency may be of global concern (59).

Folate was also consumed at an insufficient level in the group of conventional orchardists. Folate is an essential dietary component that affects cognitive function, cancer, cardiovascular disease or prevents neural tube defects (NTDs) (60). Adequate folate intake is therefore particularly important among women of childbearing age. Although the average age in the study group is 44 ± 8 years, some of the study group is even under 30 years of age. Particular attention to adequate folate intake is therefore important.

It is worth noting that organic orchardists consumed higher amounts of potassium, calcium, phosphorus, magnesium, iron, zinc, copper and manganese, as well as vitamins: E, C, B2, B6, and folate. The lack of significant differences in energy and macronutrient intake indicates that the organic fruit growers' diets must have been more varied and the nutrition density of diet was higher in this group. However, it is difficult to make a final assessment of the intake of selected vitamins and minerals because the current tables of composition and nutritional value of products and foods (61), upon which the dietetic software available in Poland is based, does not distinguish between organic and conventional products and their composition. It is additionally worth noting that organic products differ in composition from conventional products. As studies have shown, organic products not only contain more antioxidants (11–16), but also calcium (62–67), iron (17, 19, 68, 69), manganese (70, 71), as well as vitamins: E (19, 72, 73) and C (11, 70–72, 74), although some studies on calcium (70), iron (62, 70, 75) and vitamin C (76, 77) gave different results. Equivocal results were obtained for phosphorus (78, 79), magnesium (66, 67, 70, 78, 80), copper (66, 70), and a significant number of studies showed lower zinc (70, 71, 79) and folate content (77) in the case of organic farming. Rationally assuming that organic orchardists are more likely to consume the food they produce (6), then the content of selected minerals and vitamins is likely to be even higher. This is particularly important for nutrients whose intake of recommended values has already been exceeded. However, it is difficult to make a final estimate of how much richer the diet of organic fruit growers is in selected ingredients without first updating the Polish tables of composition and nutritional values of food products.

A higher intake of individual nutrients means that the ration contained more products as sources of these nutrients. However, this can be considered a positive development when it allows us to get closer to the recommended intake values. Otherwise, the consumption of products that are sources of those nutrients may have a neutral effect on meeting needs, or a negative effect if the recommended intake values are significantly exceeded. Phosphorus is worth noting in this context. The organic fruit growers consumed significantly more of it, but due to meeting the nutritional needs for phosphorus in both groups, they also deviated significantly more from the recommended intake values in a negative way - significantly exceeding the recommended values. A similar situation is observed for iron, manganese and vitamin B2, where a small percentage of individuals in both groups did not meet the recommended values, and organic fruit growers consuming significantly more of these nutrients with their ration also exceeded the recommended intake values significantly more. In contrast, the opposite situation is observed for folate. A significant percentage of the study group did not meet their folate requirements. The significantly lower deviation from the recommendations in the case of the organic fruit growers was a positive development, and meant that the nutritional needs were better covered and the intake was closer to the recommended values.

There is a lack of research with fruit growers on their diets and the nutrient content of their food rations. There are also no studies comparing the diets of organic and conventional orchardists. A studies by the other authors, carried out with farmers, i.e., the group most similar to our study group, also showed a low intake of calcium (81) and most nutrients (82). However, they were not conducted on a European population. It is therefore difficult to apply them to the results obtained in the present study. This does, however, highlight the need for continued research in similar groups.

The vast majority of the group consumed too much cholesterol and saturated fatty acids, while they consumed too little n-3 polyunsaturated fatty acids and n-6 linoleic acid. The structure of cholesterol and fatty acids intake in the study group is therefore strongly unfavorable from the point of view of the prevention and treatment of cardiovascular disease, where such proportions are considered to be harmful and the opposite to be health-promoting (83–86). However, it should be noted that organic foods contain higher amounts of n-3 polyunsaturated fatty acids, or CLA (17–19). Organic orchardists, therefore, by consuming the food they produce, may be able to cover their nutritional needs to a greater extent and the diet may have more cardioprotective properties. Of course, detailed studies of the composition of the diet consumed by organic fruit growers are needed to confirm or reject these hypotheses.

## 5 Limitations

This study, although carried out with the highest degree of care has some limitations. The main limitation is that the study group was relatively small - a total of 53 people, due to the large area covered and the capacity of the research team. For this reason, the study should be considered a pilot study.

Another limitation is that a single measurement was carried out using the 3-day dietary record method. The results may present a bump in intake that does not reflect the standard diet among fruit growers throughout the year. Another limitation is the period of the survey when fruit growers spent more time at home. This may carry a misconception about the amount and size of portions consumed during the year-round period, which is likely to be distinguished by different eating habits in the study group.

The study also has strengths. First and foremost, it introduces answers to questions that have not been addressed before - what is the nutritional value of the diet of orchardists. This research also has the unique value of comparing two groups with similar lifestyles, but cultivating different methods in their work, which is to some extent due to the worldview presented - organic and conventional fruit growers. In a way, this study also brings data on how diet translates into differences in health issues in these groups. However, the latter issue requires further study.

## 6 Conclusions

In conclusion, the fruit growers' food ration presented was mostly of adequate nutritional value. Mainly insufficient intake was observed for calcium and vitamin D, which may reflect on the bone health of the orchardists, and the cholesterol and fatty acids profile, which may have a bearing on the risk of cardiovascular disease.

The organic orchardists' diets were richer in nutrients, as a result of a more varied diet. Conventional orchardists additionally consumed insufficient amounts of potassium, magnesium and vitamins: E, C, and folate, which may further increase the risk of the previously mentioned cardiovascular diseases. Prevention in this regard seems fully justified. Therefore, the main recommendation arising from this research is the need for nutrition education among all orchardists, with a primary focus on conventional growers.

Further studies, preferably longitudinally, are needed to clearly identify differences in diet and food intake in the orchardists group. Future research should also focus on in-depth analysis of potential correlations between dietary deficiencies and health consequences in a group of fruit growers and farmers, further highlighting possible interventions that could improve health in this group. Research into biochemical indicators, such as blood analysis, and their relationship to diet is therefore also extremely necessary. Further, comparison of the diets of the organic and conventional groups is also warranted.

## Data availability statement

The raw data supporting the conclusions of this article will be made available by the authors, without undue reservation.

## Ethics statement

The studies involving humans were approved by Rector's Committee on Ethics in Human Research at SGGW. The studies were conducted in accordance with the local legislation and institutional requirements. The participants provided their written informed consent to participate in this study.

## Author contributions

HD: Conceptualization, Data curation, Formal analysis, Investigation, Methodology, Project administration, Resources, Supervision, Validation, Visualization, Writing – original draft, Writing – review & editing. BS: Data curation, Formal analysis, Software, Visualization, Writing – review & editing. DW: Conceptualization, Methodology, Resources, Writing – original draft, Writing – review & editing. RK: Data curation, Investigation, Resources, Writing – review & editing. JO: Data curation, Investigation, Writing – review & editing. ER: Conceptualization, Data curation, Formal analysis, Funding acquisition, Investigation, Methodology, Project administration, Resources, Supervision, Writing – original draft, Writing – review & editing.
